# Consensus Recommendations for MRD Testing in Adult B-Cell Acute Lymphoblastic Leukemia in Ontario

**DOI:** 10.3390/curroncol28020131

**Published:** 2021-03-30

**Authors:** Anne Tierens, Tracy L. Stockley, Clinton Campbell, Jill Fulcher, Brian Leber, Elizabeth McCready, Peter J. B. Sabatini, Bekim Sadikovic, Andre C. Schuh

**Affiliations:** 1 Laboratory Medicine Program, Hematopathology, University Health Network, Toronto, ON M5G 2C4, Canada; anne.tierens@uhn.ca; 2 Department of Laboratory Medicine and Pathobiology, University of Toronto, Toronto, ON M5S 1A8, Canada; Tracy.Stockley@uhn.ca (T.L.S.); peter.sabatini@uhn.ca (P.J.B.S.); 3 Laboratory Medicine Program, Division of Clinical Laboratory Genetics, University Health Network, Toronto, ON M5G 2C4, Canada; 4 Department of Pathology and Molecular Medicine, Hamilton Health Sciences, McMaster University, Hamilton, ON L8S 4L8, Canada; campbecj@mcmaster.ca (C.C.); mccready@hhsc.ca (E.M.); 5 Department of Medicine, University of Ottawa, Ottawa, ON K1H 8M5, Canada; jfulcher@toh.ca; 6 The Ottawa Hospital Research Institute, Ottawa, ON K1H 8L6, Canada; 7 Department of Medicine, McMaster University, Hamilton, ON L8S 4L8, Canada; leberb@mcmaster.ca; 8 Hamilton Regional Laboratory Medicine Programme, Hamilton Health Sciences, Hamilton, ON L8N 4A6, Canada; 9 London Health Sciences Centre, Molecular Genetics Laboratory, Molecular Diagnostics Division, London, ON N6A 5W9, Canada; bekim.sadikovic@lhsc.on.ca; 10 Department of Pathology and Laboratory Medicine, Western University, London, ON N6A 5C1, Canada; 11 Princess Margaret Cancer Centre, Division of Medical Oncology and Hematology, Toronto, ON M5G 2M9, Canada; 12 Department of Medicine, University of Toronto, Toronto, ON M5S 3H2, Canada

**Keywords:** adult B-cell acute lymphoblastic leukemia, adult acute lymphoblastic leukemia, flow cytometry, minimal residual disease, measurable residual disease, next-generation sequencing, polymerase chain reaction

## Abstract

Measurable (minimal) residual disease (MRD) is an established, key prognostic factor in adult B-cell acute lymphoblastic leukemia (B-ALL), and testing for MRD is known to be an important tool to help guide treatment decisions. The clinical value of MRD testing depends on the accuracy and reliability of results. Currently, there are no Canadian provincial or national guidelines for MRD testing in adult B-ALL, and consistent with the absence of such guidelines, there is no uniform Ontario MRD testing consensus. Moreover, there is great variability in Ontario in MRD testing with respect to where, when, and by which technique, MRD testing is performed, as well as in how the results are interpreted. To address these deficiencies, an expert multidisciplinary working group was convened to define consensus recommendations for improving the provision of such testing. The expert panel recommends that MRD testing should be implemented in a centralized manner to ensure expertise and accuracy in testing for this low volume indication, thereby to provide accurate, reliable results to clinicians and patients. All adult patients with B-ALL should receive MRD testing after induction chemotherapy. Philadelphia chromosome (Ph)-positive patients should have ongoing monitoring of MRD during treatment and thereafter, while samples from Ph-negative B-ALL patients should be tested at least once later during treatment, ideally at 12 to 16 weeks after treatment initiation. In Ph-negative adult B-ALL patients, standardized, ideally centralized, protocols must be used for MRD testing, including both flow cytometry and immunoglobulin (*Ig*) heavy chain and T-cell receptor (*TCR*) gene rearrangement analysis. For Ph-positive B-ALL patients, MRD testing using a standardized protocol for reverse transcription real-time quantitative PCR (RT-qPCR) for the *BCR-ABL1* gene fusion transcript is recommended, with *Ig/TCR* gene rearrangement analysis done in parallel likely providing additional clinical information.

## 1. Introduction

The overall incidence of acute lymphoblastic leukemia (ALL) in Canada is 0.8 per 100,000 person years. ALL incidence follows an age-dependent biphasic distribution with a peak incidence at 1 to 4 years, and a second peak at approximately age 75 [1,2]. The overall survival for adult ALL has improved significantly over the last 20 years due to advances in treatment and supportive care, but is still lagging behind pediatric ALL. The 5-year survival rate for adult ALL is now 50% or more in adults up to 50 to 60 years of age, and has also improved for older patients.

ALL is a disease that is characterized by genomic alterations that result in the abnormal differentiation and proliferation of lymphoid precursor cells. In adults, 85% of cases develop from precursors of the B-cell lineage, with the remainder having a T-cell phenotype [3]. The identification of cytogenetic and molecular abnormalities in B-ALL provides important prognostic information and helps to guide treatment decisions. The most frequent abnormality in adult ALL is the *BCR-ABL1* translocation t(9;22)(q34;q11) (Philadelphia chromosome [Ph]). Leukemias with this translocation, Ph-positive ALL, comprise about 25% of cases of adult ALL overall, but with an increasing incidence with age, reaching 40 to 50% of adult ALL cases above age 50 [4,5,6]. ALLs bearing the *BCR-ABL1* translocation have historically had a particularly poor prognosis. The development of tyrosine kinase inhibitor therapy for Ph-positive B-ALL has dramatically improved outcomes for these patients, however [7]. Similarly, while the treatment of ALL relapse has historically been mostly unsuccessful, the advent of the targeted immunotherapies, inotuzumab ozogamicin and blinatumomab, as well as of chimeric antigen receptor (CAR)-T cell therapy, has also improved outcomes for B-ALL patients with relapsed/refractory disease [8,9]. 

Over the last decade, additional subtypes of Ph-negative B-ALL have been identified [6], many of which are associated with a distinct prognosis. Identifying sub-type specific therapy for such patients is an ongoing research effort. One subtype, Ph-like (*BCR-ABL1*-like) B-ALL was originally defined as having a gene expression profile identical to that of Ph-positive ALL, but lacking the t(9;22)(q34;q11). Ph-like B-ALL peaks in adolescents and young adults, with an incidence of approximately 28% in adults, and is associated with unfavourable outcomes and a higher risk of treatment failure [10]. In addition, rearrangements of genes such as *CRLF2* and *CEBP* family members into the immunoglobulin heavy chain variable gene (IGHV gene) locus are predominantly found in adult B-ALL, and confer a poor prognosis [11]. Other B-ALL subtypes that confer a more favourable prognosis include the PAX5 D80R subtype, and *DUX4*-rearranged B-ALL [6].

Although most adults with B-ALL achieve complete morphological remission with intensive induction and consolidation therapy, 40 to 50% will ultimately relapse over time [12,13]. A strong prognostic factor for relapse is the presence of post-treatment measurable (minimal) residual disease (MRD) [14,15]. While the sensitivity of MRD detection depends on the assay used, a common operational cut-off for MRD positivity is 10^−4^ (1 leukemic cell in 10,000 cells assayed). Patients who achieve MRD negativity after induction, or at a later time point, have improved relapse-free survival as well as improved overall survival. These outcomes are noted across all subgroups, including both Ph-positive and Ph-negative patients [16]. Therefore, MRD levels not only inform decisions regarding the need for, and timing of, allogeneic hematopoietic stem cell transplantation (alloSCT), but also help guide modifications to ongoing management. In the case of alloSCT, not only do patients that are MRD-negative prior to alloSCT have better outcomes than do those with detectable MRD [17,18,19,20,21,22], but alloSCT also improves outcomes in patients that are persistently MRD positive. By extension, there is increasing evidence suggesting that patients with an optimal treatment response and MRD negativity may not need alloSCT [17,23,24]. MRD positivity at defined timepoints may also be used to guide changes in ongoing leukemia management. For example, in Ph-positive ALL, MRD testing may indicate the need to change the TKI used, while in Ph-negative ALL, persistent MRD positivity may trigger the use of MRD-directed therapies such as blinatumomab. Based on the ability of MRD testing to predict treatment outcomes post chemotherapy, antibody/BiTE, and CAR-T therapies, thereby informing alloSCT decisions, it is likely that MRD levels will soon function as surrogate outcome indicators, at least in the context of a clinical trial.

To be optimally useful clinically, MRD assessment requires accurate, reproducible, and sensitive detection of very low levels of residual leukemia. This can be done using either flow cytometry, or molecular genetic methods. Flow cytometry for MRD analysis involves identifying and tracking aberrant leukemia-associated immunophenotypes (LAIP) found on leukemic cells [25]. Molecular genetic methods include quantitative polymerase chain reaction (qPCR), droplet digital PCR (ddPCR), or next generation sequencing (NGS) to detect the presence of leukemia-specific gene fusions, such as *BCR-ABL1*, or clonal rearrangements in the immunoglobulin (*Ig*) and T-cell receptor (*TCR*) gene families [26]. 

The clinical value of MRD results depend on the accuracy and reliability of these assays. In contrast to the long-established standardized and centralized MRD approaches employed in other jurisdictions (especially in Europe) [27], MRD testing in adult ALL in Ontario is currently highly variable in terms of how and when it is performed, and this lack of consistency affects the quality of care that can be delivered to patients. There are currently no Canadian guidelines for laboratories doing MRD testing in ALL, although Cancer Care Ontario has recently published recommendations stating that MRD testing can be considered to help select between various treatment options for adults with ALL, in addition to its use as a prognostic test [28]. An expert multidisciplinary working group was convened to discuss the status of MRD testing in Ontario, and to define recommendations for improving the delivery of MRD testing in adult B-ALL. 

## 2. Working Group and Methods

An expert multidisciplinary working group meeting was held in Toronto in November, 2019. Four Ontario institutions were represented, selected for their expertise in ALL treatment and diagnosis (including MRD testing). The corresponding Hematology, Hematopathology, and Laboratory Medicine physicians/PhDs divided into subgroups within the meeting to draft recommendations on when to perform MRD testing in Ph-positive and Ph-negative B-ALL patients, and how to perform MRD testing with respect to methodology and standardization. Current evidence from the literature, as well as evidence gaps, were considered when drafting recommendations. Subgroups presented draft recommendations to the complete group and a set of consensus recommendations was created. 

## 3. Recommendations from the Working Group

### 3.1. Ensuring Quality of MRD Testing

Recommendations:

1. MRD testing in Ontario should be centralized in an accredited laboratory. Until centralization is implemented, the standardization of testing approaches and the use of common quality metrics is mandatory among labs doing MRD testing.
2. Bone marrow specimens should be used for MRD testing.
3. The most sensitive methods of MRD detection should be chosen for routine clinical use. While both flow cytometric and molecular genetic approaches can deliver sensitivities of 10^−4^, this sensitivity is more consistently delivered by molecular analysis.

MRD results must be timely, accurate and reliable, to ensure the validity of MRD-based prognostic and therapeutic decisions. Regardless of the methodology used, a certain volume of testing is necessary to achieve the required level of expertise in MRD testing. For example, accurate flow cytometry assessment is highly dependent on the expertise of the interpreting Hematopathologist [26]. In addition, assay sensitivity for a standardized reverse transcription real-time quantitative PCR (RT-qPCR) MRD assay for the *BCR-ABL1* transcript in Ph-positive ALL is related to the laboratory’s level of experience with the specific protocol [29]. Given the relatively low incidence of adult ALL, performing MRD testing in experienced centralized laboratories will provide clinicians with the most consistent and reliable results. Shipping of samples to centralized laboratories is feasible, even for flow cytometry samples, which require the sample to arrive at the laboratory for testing within 48 h. This model of centralized testing is standard practice in European countries: a survey of MRD testing patterns in France, Germany, Italy, Spain and the UK found that the majority of clinicians in those countries used centralized laboratories for testing, consistent with national protocols that recommend MRD testing to inform treatment decisions [27].

To improve the quality of MRD testing in Ontario, the working group recommends that MRD testing should be centralized. Until this is achieved, the shared use of a single, standardized, internationally validated, method by laboratories performing MRD testing is essential. Standardization of approaches and of quality metrics is required to ensure that MRD tests done at different laboratories perform equally well, that results from different laboratories can be compared, and that results are reliably and consistently interpreted. In the absence of centralization, it is only with standardization of technique that the clinical relevance and potential actionability of MRD positivity/negativity can be interpreted in a consistent manner across the province. 

All laboratories performing MRD testing must be able to reproducibly detect low levels of MRD at a threshold agreed upon for clinical relevance, and using an appropriately sensitive test. A common operational cut-off for MRD positivity is 10^−4^ [30]. This level of sensitivity is achieved routinely by molecular genetic approaches, but is not obtained routinely by flow cytometry, although some laboratories in Ontario using state-of-the-art flow cytometric techniques can achieve this, assuming that the patient sample submitted is adequate. In addition, whether MRD testing is centralized or not, EQA/proficiency testing (PT) is essential to ensure consistency in testing among laboratories. Accreditation for MRD assays in Ontario is not yet provided by IQMH, or included in the general ISO 15189 accreditation, which any laboratory performing MRD testing should have. Laboratories are also required to participate in proficiency testing initiatives for all MRD assays they perform, which could include PT programs from CAP, UKNEQAS, and EuroMRD. It is recommended that Ontario laboratories performing MRD analyses also participate in assay-specific, internationally defined quality control initiatives, and many consortia have established accreditation programs for laboratories participating in their studies. 

Guidelines for data interpretation and reporting are critical to ensure accurate results across multiple laboratories. While the interpretation of flow cytometric data is the most difficult component to standardize [31], variability in interpretation of the same data set can also occur in molecular genetic MRD testing [29]. The manner in which MRD is reported is also important, to ensure that clinicians have the information they need to guide decision-making. Both flow cytometry and molecular MRD reports should be concise to allow clinicians to draw clear conclusions. Reports should include information on the quality of the sample (when relevant), the reproducible sensitivity (lower level of quantitation) and level of detection, the MRD value, a description of the detected LAIP or molecular marker, and a conclusion, and should be in keeping with all other laboratory accreditation requirements for reporting. For molecular-based tests, MRD-positive results that are below the defined quantifiable range should also be clearly indicated if applicable. The molecular reports should also include the specific target(s) tested to ensure that the appropriate genomic analyte was ascertained for MRD analysis. A brief summary of the molecular method and its limitations should be described in the final report, as is required by Ontario laboratory accreditation requirements. 

The working group recommends that bone marrow specimens be used for MRD testing. Bone marrow specimens are preferred, as studies have shown that blood and marrow results can be highly discordant, with MRD levels up to 10^3^ times higher in the bone marrow than in the peripheral blood [32]. In addition, it is bone marrow samples that have been used historically in studies of the prognostic and predictive value of MRD testing in B-ALL, and thus there is no real evidence base supporting the use of peripheral blood samples for this purpose [14,15,16,18,33]. Nevertheless, while studies in ALL have shown that the percentage of blasts is lower in peripheral blood than in bone marrow samples analayzed in parallel, it has been suggested that peripheral blood could be used for MRD analysis, assuming that the assay is suitably sensitive, and the total number of input cells is sufficient (i.e., ≥10^6^ cells) [32,34]. This approach is not standardized, however. When bone marrow is used, it is similarly important to ensure that the input cell number is optimal. For this reason, the first aspirated marrow specimen (the ‘first pull’), with a volume of 1 to 2 mL is required for MRD testing, and this requirement is particularly important in the context of flow cytometry MRD, for which input cell number is key. For molecular MRD, genomic input minimums (rather than input cell numbers) ensure that appropriate sensitivity requirements can be achieved. For example, 1 µg of DNA corresponds to approximately 150,000 cells (assuming 6.5 pg DNA per cell) to achieve a sensitivity of 10^−5^ for *Ig/TCR* rearrangement analysis [35]. Similarly, an RNA input sufficient to produce 100,000 copies of *ABL1* by RT-qPCR would result in a sensitivity of 10^−5^ for *BCR-ABL1* detection [36].

### 3.2. MRD Testing in Ph-Negative ALL

Recommendations:

1. Adult Ph-negative ALL patients should receive MRD testing after induction chemotherapy, and at least one additional time point later in treatment, around 12–16 weeks.
2. Flow cytometry and analysis of *Ig/TCR* gene rearrangements are both acceptable approaches for MRD testing in adult Ph-negative ALL patients, provided that the laboratory can reliably meet the required sensitivity of at least 10^−4^. Standardized, accredited protocols with a validated quality assurance program must be used.

The importance of incorporating MRD assessment into routine care for Ph-negative ALL patients has been demonstrated in multiple studies. Patients with lower MRD levels after induction and consolidation chemotherapy had longer duration of remission, longer relapse-free survival, and longer overall survival [37]. MRD levels may also be used as a stratification tool to inform treatment decisions: for example, alloSCT may be avoided, while maintaining favourable outcomes, for patients with good early MRD responses [17,18]. MRD testing in first or second complete remission also informs the use of the targeted therapy blinatumomab, which is the first Health Canada-approved therapy for Ph-negative B-ALL patients with MRD greater than or equal to 0.1%. Further, patients with MRD reappearance have worse outcomes compared to those with MRD persistence, suggesting that ongoing monitoring of MRD levels is helpful for risk assessment [37]. The optimal timing of MRD assessment in adults has been studied in detail [38]. Based on these data, the working group recommends that all Ph-negative ALL patients should receive MRD testing after induction chemotherapy. At a minimum, one additional MRD test should be done around 12–16 weeks, after all drugs have been delivered at a maximal dose. There may be a need for further MRD tests in some patients depending on disease progression and characteristics. This approach aligns with current recommendations from the National Comprehensive Cancer Network in the U.S. and the European Society for Medical Oncology [39,40]. It is important to remember that the predictive value of MRD testing depends both on the specific treatment regimen employed, and on the time points used for MRD testing. The optimal time points for testing related to the therapy regimens typically used in Canada are not yet clearly defined in a protocol-specific manner, but the 12–16 week rule can be applied widely.

Currently in Ontario, MRD testing in Ph-negative B-ALL patients is done using flow cytometry, although some laboratories are developing molecular genetic testing methods. The working group recognizes that flow cytometry and *Ig/TCR* gene rearrangement analysis are both widely accepted approaches for MRD testing in Ph-negative B-ALL patients, provided that the minimum sensitivity requirement of 10^−4^ is met by a standardized protocol (Table 1). These approaches are not fully interchangeable, however, as they may provide complementary information in some clinical contexts, such as post-induction. Notably, while both methods can provide high sensitivity detection of MRD at a level of 10^−4^, this is more likely achievable on a routine basis using a molecular approach. In general, molecular methods also allow for greater interlaboratory comparability than do flow cytometry-based methods [41]. Standardized protocols have been developed and used successfully in Europe for both technologies, however [42,43]. Standardization of methodologies reduces inter-laboratory variability, minimizes the rates of false positive and false negative results, allows for the optimization of protocols, and creates consistency in interpretation and reporting of results. Application of standardized protocols to MRD testing in Ontario will improve the quality of testing and will provide clinicians with more reliable results to guide clinical decision making. 

Flow cytometry is currently the most widely used technique in North America for assessing MRD in Ph-negative B-ALL patients. Modern flow cytometry techniques using 6 or more fluorochromes are capable of detecting MRD with a sensitivity of approximately 10^−4^ (assuming an adequate sample), and there is the potential for even greater sensitivity with more than 8 fluorochromes and a higher number of input cells, although such sensitivity is not achieved routinely [26]. MRD assessment by flow cytometry is affordable and has a quick turnaround time. However, the interpretation of flow cytometry results for MRD assessment requires substantial expertise from the Hematopathologist [26]. Currently, Ontario laboratories performing flow cytometry for MRD assessment in adult B-ALL are not all using standardized protocols. Several different consortia have published standardized methods with external quality assurance programs, including the EuroFlow Consortium, the AIEOP-BFM Consortium, the Children’s Oncology Group and the NOPHO group [31,43,44,45]. The working group recommends that MRD assessment by flow cytometry in Ontario should be centralized, and that the laboratory performing testing must use one of these standardized protocols. The laboratory should provide flow cytometry results in less than 48 h. 

Although molecular methods for MRD assessment in Ph-negative B-ALL patients are widely used in Europe, these methods still require development in Canada. The use of real-time quantitative PCR (RQ-PCR) to identify and follow *Ig/TCR* rearrangements has been standardized by the EuroMRD consortium, and although this method is highly sensitive, it is labour intensive and requires a reference diagnostic sample to define patient-specific primers and to use as a quantification stardard [32]. Recent progress has been made by the EuroMRD group to develop, validate, and standardize NGS assays to evaluate *Ig/TCR* rearrangements for MRD assessment in ALL [42]. Although this method does not require the use of patient-specific probes, it does require a diagnostic sample to identify the leukemia-associated *Ig/TCR* rearrangement. Sensitivity of this method varies based on the amount of input DNA: a sensitivity of 10^−4^ to 10^−5^ can be reached with 500 ng to 1 μg of input DNA. In comparison with flow cytometry and RQ-PCR, NGS generally shows better sensitivity [26]. However, the quantification of MRD levels by NGS can be challenging. Given the advantages of NGS compared to RQ-PCR for MRD detection, and its increased adoption and standardization in Europe, as Canadian laboratories develop molecular assays for MRD detection in Ph-negative ALL, the working group recommends that molecular MRD testing be performed in a central laboratory in Ontario. Ideally, an NGS-based assay should be used, with a standardized lab-developed and validated protocol such as the EuroClonality-NGS assay. However, a hybrid NGS/RQ-PCR approach may currently be more feasible, in which NGS is used to identify PCR MRD targets, which subsequently can be used to monitor MRD with the well-established patient-specific RQ-PCR method. In situations where no *Ig/TCR* rearrangement can be identified due to limitations of PCR reactivity, flow cytometry should be performed as an alternative. Turnaround time for molecular MRD testing results should ideally be within 10 calendar days, to ensure results are received in a clinically relevant time frame. However, recognizing that achievement of this goal will require additional resources and funding to be provided to laboratories, the maximum acceptable turnaround time in the current laboratory environment is 14 calendar days, with the optimal goal of returning results to the clinician within 10 calendar days. In contrast, the turnaround time for flow cytometric MRD assessment will be much shorter.

### 3.3. MRD Testing in Ph-Positive ALL

Recommendations:

1. Adult Ph-positive ALL patients should receive MRD testing after induction chemotherapy, with ongoing monitoring thereafter.
2. At a minimum, MRD testing for Ph-positive ALL should be centralized in a laboratory using reverse transcription real-time quantitative PCR for both the p210 and p190 *BCR-ABL1* transcripts, using standardized assays. MRD assessment using RQ-PCR/NGS assays evaluating *Ig/TCR* rearrangements should ideally also be used in parallel for Ph-positive patients, as this approach may provide additional, complementary clinical information.

Ph-positive ALL has an aggressive clinical course, with a high risk of relapse despite progress in treatments. As with Ph-negative ALL, MRD testing is useful for prognosis and management of patients with Ph-positive B-ALL. MRD negativity at three months after chemotherapy plus TKI therapy is associated with improved relapse-free and overall survival [46,47]. In addition, the evaluation of MRD assists in making decisions regarding alloSCT, and in directing modifications to ongoing treatment. Growing lines of evidence indicate that patients with deep MRD responses to initial therapy may not require alloSCT, but rather can be treated with ongoing chemotherapy plus TKI, with good outcomes [48]. In patients undergoing alloSCT, measurable MRD levels at the time of transplant are associated with a significantly higher risk of post-alloSCT relapse than observed in MRD-negative patients [19,49,50]. Ongoing MRD testing also informs the need for *ABL* mutation analysis and potential mutation-guided changes in TKI therapy, such as switching from imatinib to dasatinib, or to ponatinib. Therefore, the working group recommends that all Ph-positive ALL patients should receive MRD testing after induction chemotherapy, and ongoing monitoring thereafter. This recommendation aligns with Cancer Care Ontario and international guidelines on MRD monitoring in Ph-positive disease. For example, ESMO and NCCN guidelines both mandate MRD assessment post-induction, and periodic monitoring thereafter, including post-SCT [39,40]. Ideally, Ph-positive patients should have MRD assessment by flow cytometry after induction, and ongoing monitoring thereafter by molecular testing (Table 1). 

The working group recommends that molecular MRD testing for Ph-positive ALL should be centralized in a laboratory using reverse transcription real-time quantitative PCR (RT-qPCR) for both the p210 and p190 *BCR-ABL1* transcripts [29,51]. If centralization is not feasible, then standardization of the RT-qPCR protocol between labs should be ensured, such as in the Europe Against Cancer Program [52]. Turnaround time, as with MRD testing for Ph-negative ALL, should be no longer than 10 calendar days to allow for rapid treatment decisions. Although many laboratories in Canada routinely perform PCR assays for the p210 transcript in CML from peripheral blood, optimal MRD testing for Ph-positive patients in ALL requires the use of bone marrow samples. The advantages of RT-qPCR include high sensitivity of 10^−4^ to 10^−5^, and a rapid turnaround time [29,53]. Using the *BCR-ABL1* fusion as a marker for MRD in Ph-positive B-ALL patients is more efficient and less labour-intensive than is RQ-PCR detection of Ig/TCR gene rearrangements, which requires patient-specific probes. Nevertheless, the RQ-PCR detection of rearrangements of Ig/TCR genes, highly standardized and widely used in Europe [54], is in development in Canada, and may be clinically useful in Ph-positive ALL as well (see below). Use of this method requires a diagnostic patient sample. *Ig/TCR* gene rearrangement monitoring and flow cytometry are also suitable for patients with variant *BCR-ABL1* breakpoints, where MRD testing using p210 or p190 specific qPCR methods cannot be used. 

Detection of MRD in Ph-positive patients using both *Ig/TCR* rearrangement and *BCR-ABL1* fusion transcript detection should be considered, as it has been shown that discordance can occur between the two methods. A false positive (or negative) MRD result could influence important treatment decision-making, such as the decision to proceed to alloSCT. In both pediatric and adult studies, discordance has been reported in more than 20% of cases between positive MRD results from *BCR-ABL1* RT-qPCR, and negative MRD results from *Ig/TCR* rearrangement RQ-PCR (the latter indicates the absence of a leukemic clone). In such ‘MRD discordant’ cases, *BCR-ABL1* positivity was found to reside in non-ALL B-lymphocytes, T-cells, and/or myeloid cells, suggesting that a multipotent hematopoietic progenitor cell was the source of the translocation [55,56], resulting in a more CML-like clinical picture. In contrast, and consistent with this interpretation, in ‘MRD concordant’ cases, *BCR-ABL1* positivity was found only in ALL B-cell precursors, suggesting that the source of the translocation was a later, more-restricted precursor cell. Using both assays to monitor MRD would ensure that *BCR-ABL1* positivity not representing true residual disease does not lead to intensification of therapy that may not be necessary. Whether the requirements for alloSCT differ between MRD ‘concordant’ and ‘discordant’ adult ALL cases, as has been suggested in a pediatric series [57], remains unknown. 

Going forward, as discussed above for Philadelphia-negative ALL, current methods for detection of *Ig/TCR* rearrangements, may soon be replaced by NGS-based methods, for which standardized protocols are already available. In addition, the current RT-qPCR approach to *BCR-ABL1* transcript quantitation may become supplanted by more sensitive droplet digital PCR (ddPCR)-based approaches [58,59]. The clinical utility of the latter approach remains undefined at present, however. 

## 4. Conclusions

Consistent, reproducible, and accurate MRD testing is required for optimal management of adult B-cell ALL. Technologies for MRD testing are evolving, with the development of standardized protocols for high sensitivity flow cytometry, for RT-qPCR (or ddPCR) analysis of the *BCR-ABL1* transcript, and for PCR or NGS to evaluate *Ig/TCR* rearrangement. There are no published guidelines for Canadian laboratories regarding MRD testing in adult B-cell ALL, and as a result, testing methods and quality are highly variable. The Ontario expert working group recommends that MRD testing in Ontario should be centralized, as this allows for the necessary development of expertise in MRD testing and provides consistent, reliable results to clinicians. Standardized and accredited protocols for MRD testing must be used. Optimal care of adult B-cell ALL patients depends on accurate MRD testing, which would benefit from implementation of the recommendations described herein.

## Figures and Tables

**Table 1:** Summary of methods currently used for MRD testing in adult B-ALL.

Method	Sensitivity	Applicability
Flow cytometry	10^−3^–10^−4^	Ph-negative B-ALL Ph-positive B-ALL
RQ-PCR of *Ig/TCR* rearrangements	10^−4^–10^−5^	Ph-negative B-ALL Ph-positive B-ALL
RT-qPCR of *BCR-ABL1* transcripts	10^−4^–10^−5^	Ph-positive B-ALL
NGS analysis of *Ig/TCR* rearrangements	10^−4^–10^−5^	Ph-negative B-ALL Ph-positive B-ALL

## Data Availability

No new data were created or analyzed in this study. Data sharing is not applicable to this article.
